# METTL3-driven m^6^A modification of NLRC5 promotes renal fibrosis in chronic kidney disease through Keap1/Nrf2/ARE signaling pathway

**DOI:** 10.3389/fimmu.2026.1739011

**Published:** 2026-03-11

**Authors:** Mingzhi Xu, Ruman Chen, Xin Zeng, Mingjiao Pan, Chunli Wang, Yonghui Qi, Na An, Yafei Bai

**Affiliations:** Blood Purification Center of Hainan General Hospital, Hainan Affiliated Hospital of Hainan Medical University, Haikou, Hainan, China

**Keywords:** Keap1/Nrf2/ARE pathway, M6A RNA methylation, METTL3, NLRC5, renal fibrosis

## Abstract

**Background:**

METTL3-mediated m^6^A RNA methylation has been implicated in renal fibrosis, a central pathological feature of chronic kidney disease (CKD). NLRC5, the largest NLR family member, is a direct m^6^A target of METTL3, but its role in METTL3-driven renal fibrosis remains unclear.

**Methods:**

An *in vitro* renal fibrosis model was established using TGF-β1–stimulated human proximal tubular (HK-2) cells. METTL3-mediated m^6^A modification and stabilization of NLRC5 mRNA were assessed by m^6^A quantification, RNA stability, MeRIP, and RIP assays. Functional impacts on the Keap1/Nrf2/ARE pathway and fibrotic responses were examined using METTL3 inhibition (STM2457, 10 μM), NLRC5 knockdown or overexpression, Keap1 overexpression, and Nrf2 inhibition (ML385, 5 μM). Fibrotic markers, inflammatory cytokines (IL-1β, TNF-α; ELISA), and oxidative stress (ROS/DCF-DA, SOD, MDA) were measured. NLRC5-overexpression effects on the Keap1/Nrf2/ARE pathway were additionally evaluated. *In vivo* validation employed a unilateral ureteral obstruction (UUO) mouse model, with kidney injury and fibrosis assessed via H&E, Masson’s staining, IHC, ELISA, Western blot, and qRT-PCR.

**Results:**

TGF-β1 upregulated METTL3, NLRC5, and global m^6^A levels in HK-2 cells. METTL3 directly bound and stabilized NLRC5 mRNA via m^6^A modification. METTL3 overexpression exacerbated TGF-β1-induced inflammation, oxidative stress, and fibrosis, which were reversed by STM2457. Conversely, METTL3 or NLRC5 inhibition suppressed fibrosis, coinciding with Keap1 downregulation and Nrf2/HO-1/NQO1 upregulation. Keap1 overexpression negated the anti-fibrotic effects of NLRC5 knockdown, while NLRC5 overexpression decreased nuclear Nrf2 and downstream antioxidant targets, confirming NLRC5’s inhibitory role on Keap1/Nrf2 signaling. Nrf2 inhibition (ML385) or NLRC5 overexpression rescued METTL3 knockdown phenotypes. *In vivo*, METTL3 knockdown attenuated UUO-induced renal injury and fibrosis, activating the Keap1/Nrf2/ARE pathway.

**Conclusions:**

METTL3 promotes renal fibrosis by stabilizing NLRC5 mRNA via m^6^A modification, leading to suppression of the protective Keap1/Nrf2/ARE pathway. Targeting the METTL3/NLRC5/Keap1/Nrf2/ARE axis may represent a promising therapeutic strategy for CKD-associated fibrosis.

## Introduction

Chronic kidney disease (CKD), characterized by the progressive decline of renal function and structural integrity, has emerged as a major global health burden, affecting nearly one in ten adults worldwide (1, 2). Regardless of the initial cause, persistent renal injury ultimately leads to renal fibrosis, the final common pathway of CKD, which is marked by excessive extracellular matrix (ECM) accumulation and irreversible tissue remodeling (3, 4). The development of renal fibrosis involves complex interactions among multiple cell types and signaling pathways (5, 6). As the predominant cell type within the renal parenchyma, tubular epithelial cells are highly susceptible to injury and undergo a range of maladaptive alterations—such as shifts in metabolism, partial epithelial-to-mesenchymal transition (EMT), and premature senescence—that collectively accelerate the fibrotic process (4, 7, 8). Increasing evidence indicates that oxidative stress is a critical driver of these pathological changes, with the Keap1/Nrf2/ARE signaling pathway playing a pivotal role in maintaining redox balance and modulating fibrotic responses (9–12).

The Keap1/Nrf2/ARE pathway serves as a central cellular defense mechanism against oxidative and electrophilic stress (9). Under normal conditions, Keap1 binds Nrf2 and promotes its ubiquitination and proteasomal degradation (13). Upon oxidative challenge, conformational changes in Keap1 inhibit Nrf2 degradation, allowing its nuclear translocation and subsequent activation of ARE-driven genes involved in antioxidant defense, detoxification, and anti-fibrotic processes (14). Dysregulation of this pathway has been increasingly linked to CKD and renal fibrosis, where sustained oxidative stress and impaired Nrf2 activation exacerbate epithelial injury and matrix deposition (10). Recent studies have also highlighted NLRC5, the largest member of the nucleotide-binding domain and leucine-rich repeat (NLR) protein family, as a critical regulator of fibrogenesis (15, 16). Knockdown of NLRC5 has been shown to suppress renal fibroblast activation through modulation of the TGF-β1/Smad signaling pathway (17), and to mitigate *in vitro* renal ischemia-reperfusion injury via activation of the PI3K/Akt pathway (18). Furthermore, NLRC5 may interact with oxidative stress pathways, influencing fibrotic remodeling by regulating inflammatory cytokine transcription and cellular redox balance (19, 20). These findings suggest that NLRC5 as a potential mediator linking immune regulation, oxidative stress, and fibrosis in the kidney.

RNA N6-methyladenosine (m6A) modification, the most prevalent internal chemical modification of eukaryotic mRNA, has emerged as a critical mechanism for post-transcriptional gene regulation by affecting RNA stability, splicing, translation efficiency, and subcellular localization (21, 22). This modification is dynamically regulated by three classes of proteins: “writers” (methyltransferases) that catalyze m6A deposition, “erasers” (demethylases) that remove m6A marks, and “readers” (binding proteins) that interpret m6A signals to influence RNA fate (22, 23). Among the writers, METTL3 is the core methyltransferase responsible for catalyzing the majority of m6A modifications on target transcripts, thereby controlling RNA metabolism and downstream cellular processes (24). Notably, studies in endometrial cancer have demonstrated that METTL3 promotes m6A modification of NLRC5 and suppresses its degradation, revealing a post-transcriptional mechanism by which METTL3 regulates NLRC5 expression (25). Considering the established roles of NLRC5 in modulating inflammation and oxidative stress, it is conceivable that METTL3-mediated m6A modification may similarly affect the Keap1/Nrf2/ARE pathway in renal cells, thereby impacting redox homeostasis, epithelial injury, and fibrotic progression in CKD.

Despite the recognized involvement of oxidative stress, Keap1/Nrf2/ARE signaling, and m6A-mediated regulation in CKD and renal fibrosis, the potential interplay between METTL3, NLRC5, and this pathway in the kidney remains unexplored. In the present study, we investigate whether METTL3-driven m6A modification of NLRC5 regulates the Keap1/Nrf2/ARE signaling axis and contributes to renal fibrosis. Elucidating this regulatory mechanism may provide novel insights into CKD pathogenesis and identify potential therapeutic targets for mitigating renal fibrosis.

## Materials and methods

### Establishment the TGF-β1-induced cell fibrosis model

Human proximal tubular epithelial cells (HK-2, Cat. #CRL-2190) were obtained from the American Type Culture Collection (ATCC, Manassas, VA, USA) and cultured in DMEM/F12 (1:1; Grand Island, NY, USA) supplemented with 10% FBS, 1% penicillin (100 U/mL), and streptomycin (100 μg/mL) (Life Technologies, Carlsbad, CA, USA). Cells were maintained at 37 °C in a humidified atmosphere containing 5% CO_2_. For fibrosis induction, HK-2 cells (1 × 10^5^ cells/well) were seeded in six-well plates and treated with recombinant human TGF-β1 (Sigma-Aldrich) at concentrations of 1, 5, or 10 ng/mL for 12, 24, or 48 h. Expression levels of α-SMA (a marker of myofibroblast activation), Collagen I (a fibrosis-associated extracellular matrix protein), and E-cadherin (an epithelial-to-mesenchymal transition marker) were assessed to validate the *in vitro* fibrosis model.

### Quantification of m^6^A levels in total RNA

Total RNA was extracted from HK-2 cells, with or without TGF-β1 treatment, using TRIzol™ Reagent (Invitrogen), and RNA concentration and purity were assessed with a NanoDrop spectrophotometer (Thermo Fisher Scientific). The global m^6^A methylation level of total RNA was measured using the EpiQuik™ m^6^A RNA Methylation Quantification Kit (Colorimetric; Epigentek) following the manufacturer’s protocol. Absorbance at 450 nm was measured, and m^6^A content was calculated based on the standard curve provided.

### Cell transfection and treatment

Small interfering RNAs targeting METTL3 (si-METTL3), NLRC5 (si-NLRC5), and a negative control (si-NC) were purchased from GenePharma (Shanghai, China). For gene overexpression, the coding sequences of METTL3, NLRC5, and Keap1 were cloned into the pcDNA3.1 vector (Invitrogen, Carlsbad, USA) to generate OE-METTL3, OE-NLRC5, and OE-Keap1 constructs, respectively. HK-2 cells were seeded in six-well plates and transfected with either siRNAs or recombinant plasmids using Lipofectamine 3000 (Invitrogen, USA) according to the manufacturer’s instructions. After 48 h, cells transfected with OE-METTL3 were treated with the METTL3 inhibitor STM2457 (10 μM, Sigma-Aldrich), as described by Jung et al. (26), which effectively inhibits METTL3 without cytotoxicity in HK-2 cells, whereas si-METTL3 or si-NLRC5 cells were treated with the Nrf2 inhibitor ML385 (5 μM, Sigma-Aldrich), based on previous studies demonstrating effective Nrf2 inhibition without compromising cell viability (27, 28). All groups were subsequently exposed to TGF-β1 (10 ng/mL) for 48 h to induce fibrosis.

### RNA stability assay

HK-2 cells transfected with OE-METTL3 or si-METTL3 were treated with actinomycin D (5 μg/mL; HY-17559, MedChem Express, Monmouth Junction, NJ) for 0, 4, and 8 h to inhibit transcription. Total RNA was then extracted and subjected to reverse transcription. NLRC5 mRNA stability was assessed by quantifying its expression at each time point using quantitative reverse transcription PCR.

### m^6^A RNA methylation immunoprecipitation RIP and RNA RIP assays

MeRIP assays were conducted using the RNA-Binding Protein Immunoprecipitation Kit (Cat. #17–700, Millipore) according to the manufacturer’s instructions. Total RNA extracted from HK-2 cells was immunoprecipitated with an anti-m^6^A antibody (Cat. #68055–1-Ig, Proteintech, Wuhan, China) to detect m^6^A modifications on NLRC5 mRNA. For the RIP assay, HK-2 cell lysates were incubated with an anti-METTL3 antibody (Cat. #DF12020) to examine the binding of METTL3 to NLRC5 transcripts. Immunoprecipitated RNA was purified and subjected to quantitative reverse transcription PCR for NLRC5 detection. Normal IgG was used as a negative control.

### Measurement of ROS levels

Intracellular ROS levels in HK-2 cells were assessed using the DCFDA/H2DCFDA Cellular ROS Assay Kit (ab113851, Abcam). Cells were seeded in 6-well plates at a density of 3 × 10^5^ cells/well and subjected to the indicated treatments. After washing with PBS, they were incubated with 5 µmol/L 2′,7′-dichlorofluorescin diacetate (DCFDA) at 37 °C for 30 min in the dark. Fluorescence was detected using an Array Scan VII microscope (BX53, Japan), and the mean fluorescence intensity was quantified.

### Animal experiments

Male C57BL/6J mice (n = 32, 20–23 g) were obtained from the Henan Skabes Biotechnology Co., Ltd. (Henan, China). Mice were housed in a pathogen-free environment under a 12-h light/dark cycle at 22 ± 2 °C, with free access to standard chow and water. At 10 weeks of age, renal interstitial fibrosis (RIF) was induced by unilateral ureteral obstruction (UUO) as previously described (26). In brief, mice were anesthetized with intraperitoneal sodium pentobarbital (50 mg/kg), and the left ureter was exposed via a left flank incision and double-ligated with 5–0 silk sutures. Sham-operated mice underwent the same surgical procedure without ureteral ligation. To evaluate the role of METTL3 *in vivo*, mice received a direct injection of adenovirus carrying METTL3 shRNA (Ad-shMETTL3) or scrambled shRNA (Ad-shNC) into the left kidney cortex (20 µL, 3.5 × 10^8^ TU/mL; Ribobio, Guangzhou, China) using a 30-gauge needle under anesthesia. The injection was performed one week prior to UUO surgery to ensure sufficient gene silencing. Mice were then randomly assigned into four groups (n = 8 per group): sham, UUO, UUO + Ad-shNC, and UUO + Ad-shMETTL3. The sample size was based on previous study (29), which used similar numbers to achieve reliable statistical power. At 12 weeks of age (two weeks post-UUO), mice were euthanized by CO_2_ inhalation using a gradual-fill method (30–70% chamber volume per minute) followed by cervical dislocation. Blood was collected via cardiac puncture, and kidneys were harvested. One portion of kidney tissue was fixed in 4% paraformaldehyde for histological and immunohistochemical analysis, while the remainder was snap-frozen in liquid nitrogen and stored at –80°C for further molecular and biochemical assays. All animal experiments were conducted in compliance with the ARRIVE guidelines and approved by the Ethics Committee of the Hainan Affiliated Hospital of Hainan Medical University (Hainan, China).

### Biochemistry analysis

The obtained blood samples were subjected to centrifugation at 5000 g to separate the serum. Serum levels of blood urea nitrogen (BUN) and creatinine (Scr) were measured using an automated biochemical analyzer (VITROS 950, Johnson & Johnson, NJ, USA) following the manufacturer’s protocol.

### ELISA assay

Pro-inflammatory cytokines IL-1β and TNF-α were measured in the supernatant of cultured HK-2 cells and mouse serum using species-specific ELISA kits (IL-1β: #CSB-E08053h for human, #CSB-E08054m for mouse; TNF-α: #CSB-E04740h for human, #CSB-E04741m for mouse; Cusabio Biotech, Wuhan, China), in accordance with the manufacturer’s protocols.

### Determination of MDA and SOD

Malondialdehyde (MDA, #A003-1-2) and superoxide dismutase (SOD, #A001-3-2) levels in the supernatant of cultured HK-2 cells and mouse serum were quantified using commercial assay kits from Nanjing Jiancheng Bioengineering Institute, following the manufacturer’s instructions.

### Histopathological evaluation

Formalin-fixed kidneys were paraffin-embedded, sectioned at 4 μm, deparaffinized in xylene, and rehydrated through a graded ethanol series. Hematoxylin and eosin (H&E) staining was performed using a commercial kit (Cat. No. G1120, Solarbio, Beijing, China) to assess renal injury. Tubular dilation was scored semi-quantitatively based on the proportion of affected area: <25%, 25–50%, 50–75%, and 75–100%, corresponding to scores of 1 to 4, respectively. For fibrosis assessment, the sections were stained with Masson’s trichrome using a commercial kit (Cat. No. DC0032, Leagene Biotechnology, China) following the manufacturer’s instructions. Fibrotic areas were quantified using the Image-Pro Plus software (version 6.0), and the percentage of positively stained area was calculated.

### Immunohistochemical analysis

Paraffin-embedded kidney sections were deparaffinized in xylene and rehydrated through a graded ethanol series (100%, 95%, 85%, and 75%), followed by rinsing in distilled water. Endogenous peroxidase activity was quenched with 3% hydrogen peroxide. After blocking with goat serum for 1 h at room temperature, sections were incubated overnight at 4 °C with a primary antibody against α-SMA (1:200, Cat. No. 55135-1-A, Proteintech). The next day, slides were incubated with a horseradish peroxidase-conjugated secondary antibody for 1 h at 37 °C, followed by hematoxylin counterstaining. Staining was quantified as the percentage of positively stained area using Image-Pro Plus software (version 6.0).

### Quantitative reverse transcription PCR

Total RNA was extracted from cell lysates and kidney tissues using TRIzol reagent (ThermoFisher, CA, USA). Complementary DNA (cDNA) was synthesized from 1 μg of RNA using the Reverse Transcription System kit (Promega, Madison, WI, USA). Quantitative PCR was performed on a CFX96 Real-Time Detection System (Bio-Rad, Hercules, CA, USA) with SYBR Green reagents (Takara Bio Inc., Tokyo, Japan) to assess the mRNA expression of METTL3 and NLRC5. Expression levels were normalized to GAPDH, and relative quantification was calculated using the 2-ΔΔCT method. Primer sequences used are listed in **Table 1**.

**Table 1:** Primers for quantitative reverse transcription PCR.

Gene	Forward (5’ - 3’)	Reverse (5’ - 3’)
Human METTL3	TAGGATGTCGGACACGTGGA	GAGACAATGCTGCCTCTGGA
Human NLRC5	CAGGCTGGGAAGACACTCAG	TGGGCGCAGACTTCTAACAG
Human GAPDH	GGAAGGTGAAGGTCGGAGTC	TCGCCCCACTTGATTTTGGA
Mouse METTL3	AAACAGCTGGACTCGCTTCG	GAGATGGCAAGACGGATGGAA
Mouse GAPDH	CTTCTCCTGCAGCCTCGT	ATGAAGGGGTCGTTGATGGC

### Western blot analysis

Total protein from cultured cells and kidney tissues was extracted using RIPA lysis buffer (Beyotime Biotechnology, Shanghai, China). Nuclear proteins were isolated with a Nuclear and Cytoplasmic Protein Extraction Kit as previously described (30). Protein concentrations were determined using a BCA Protein Assay Kit. Equal amounts (30 μg) of protein were separated by SDS-PAGE and transferred onto PVDF membranes (Bio-Rad, Hercules, CA, USA). After blocking with 5% non-fat milk in TBST for 2 h at room temperature, membranes were incubated overnight at 4 °C with primary antibodies targeting α-SMA (1:1000, #19245), Collagen I (1:1000, #72026), E-cadherin (1:1000, #3195), Nrf2 (1:1000, #12721), Keap1 (1:1000, #8047), HO-1 (1:1000, #70081), NQO1 (1:1000, #62262), METTL3 (1:1000, #86132), NLRC5 (1:1000, #72379), Lamin B1 (1:1000, #13435), Lamin B1 (1:1000, #13435) and GAPDH (1:5000, #5174) (all from Cell Signaling Technology unless otherwise stated; dilution 1:1000, GAPDH 1:5000). After washing with TBST, membranes were incubated with HRP-conjugated secondary antibodies (Beyotime Biotechnology) for 2 h at room temperature. Protein bands were detected using an enhanced chemiluminescence (ECL) kit, and signal intensities were quantified using ImageJ software.

### Statistical analysis

Data were analyzed using GraphPad Prism version 8.0 and are presented as mean ± standard deviation (SD). Comparisons between two groups were performed using unpaired Student’s t-test, while multiple group comparisons were assessed by one-way ANOVA followed by Dunnett’s or Tukey’s *post hoc* tests, as appropriate. A *p*-value < 0.05 was considered statistically significant.

## Results

### Upregulation of m^6^A methylation and NLRC5 expression in TGF-β1–induced fibrotic HK-2 cells

**Figure 1:**
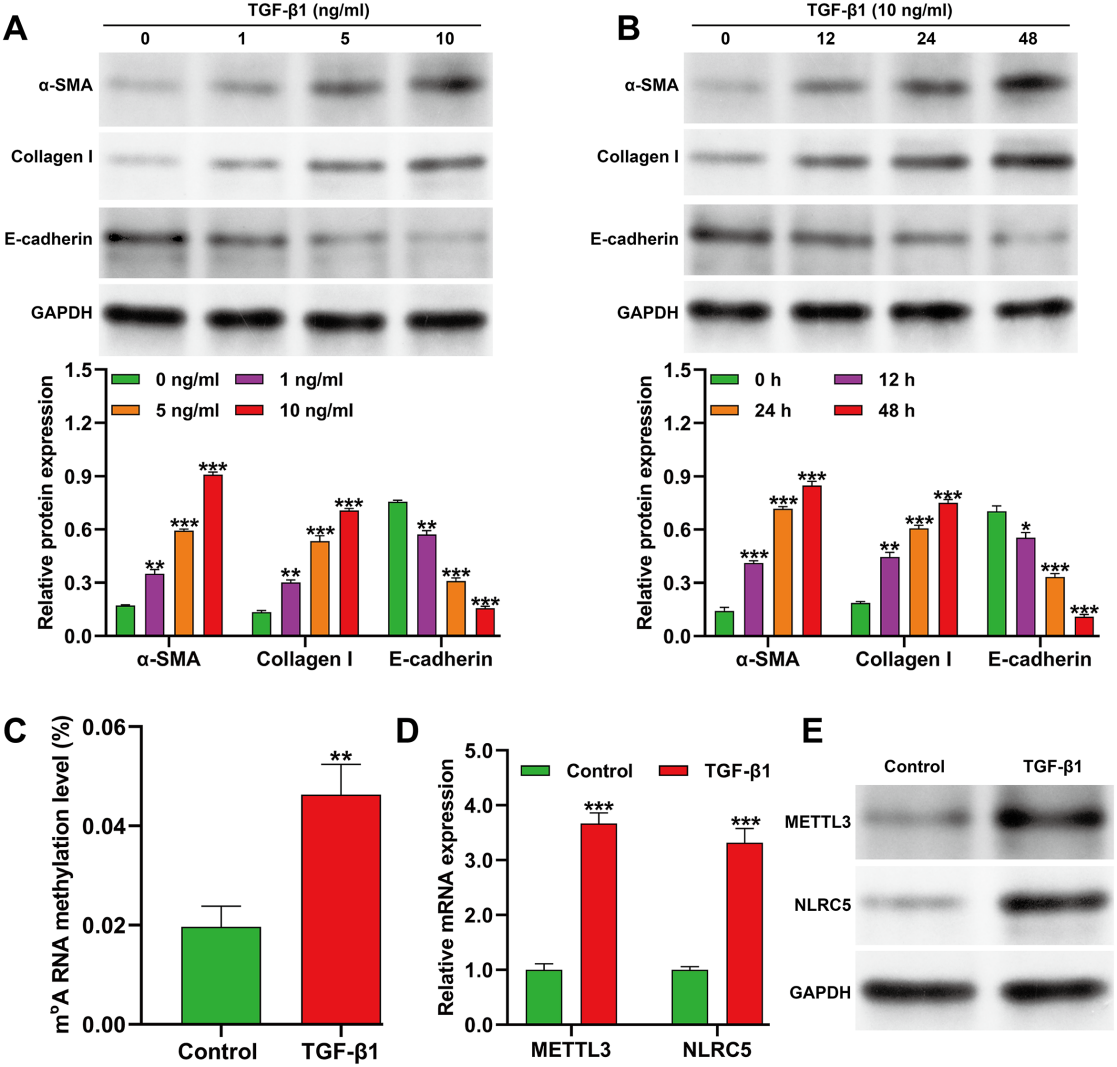
Analysis of m^6^A RNA methylation and NLRC5 expression in the TGF-β1-induced cell fibrosis model. **(A)** Western blot showing dose-dependent changes in fibrosis markers after 48 h treatment with increasing TGF-β1 concentrations (0, 1, 5, 10 ng/mL). **(B)** Time-course Western blot analysis of HK-2 cells exposed to 10 ng/mL TGF-β1 for 12, 24, and 48 (h) *p < 0.05, **p < 0.01, ***p < 0.001, *vs.* 0 ng/ml or 0 h; **(C)** Quantification of global m^6^A RNA methylation in HK-2 cells treated with 10 ng/mL TGF-β1 for 48 h, demonstrating a significant increase versus control (**p < 0.01, *vs.* control). **(D)** Quantitative reverse transcription PCR quantitative reverse transcription PCR analysis of METTL3 and NLRC5 mRNA levels after 48 h TGF-β1 treatment, indicating significant upregulation (***p < 0.001, *vs.* control). **(E)** Representative Western blots depicting METTL3 and NLRC5 protein expression in control and TGF-β1-treated cells (10 ng/mL, 48 h). Data are presented as mean ± SD from three independent experiments.

To characterize RNA methylation dynamics during renal fibrosis, a TGF-β1–stimulated HK-2 cell model was established. Fibrogenic activation was confirmed by Western blot analysis of α-SMA, Collagen I, and E-cadherin expression. As shown in **Figure 1A**, increasing TGF-β1 concentrations led to a dose-dependent elevation of α-SMA and Collagen I, accompanied by a reduction in E-cadherin. The most pronounced changes were observed at 10 ng/mL TGF-β1. Time-course experiments using this concentration further demonstrated progressive fibrosis, with maximal effects evident after 48 h (**Figure 1B**). Global m^6^A RNA methylation levels were markedly increased following TGF-β1 exposure (**Figure 1C**). Consistently, both quantitative reverse transcription PCR (**Figure 1D**) and Western blot analyses (**Figure 1E**) showed significant upregulation of the methyltransferase METTL3 and NLRC5 expression. These findings indicate that TGF-β1–induced fibrotic responses are accompanied by enhanced m^6^A RNA methylation and elevated NLRC5 expression, implying a potential epigenetic link between m^6^A modification and fibrosis progression.

### METTL3 modulated m^6^A modification and regulated NLRC5 expression in TGF-β1–induced fibrotic HK-2 cells

**Figure 2:**
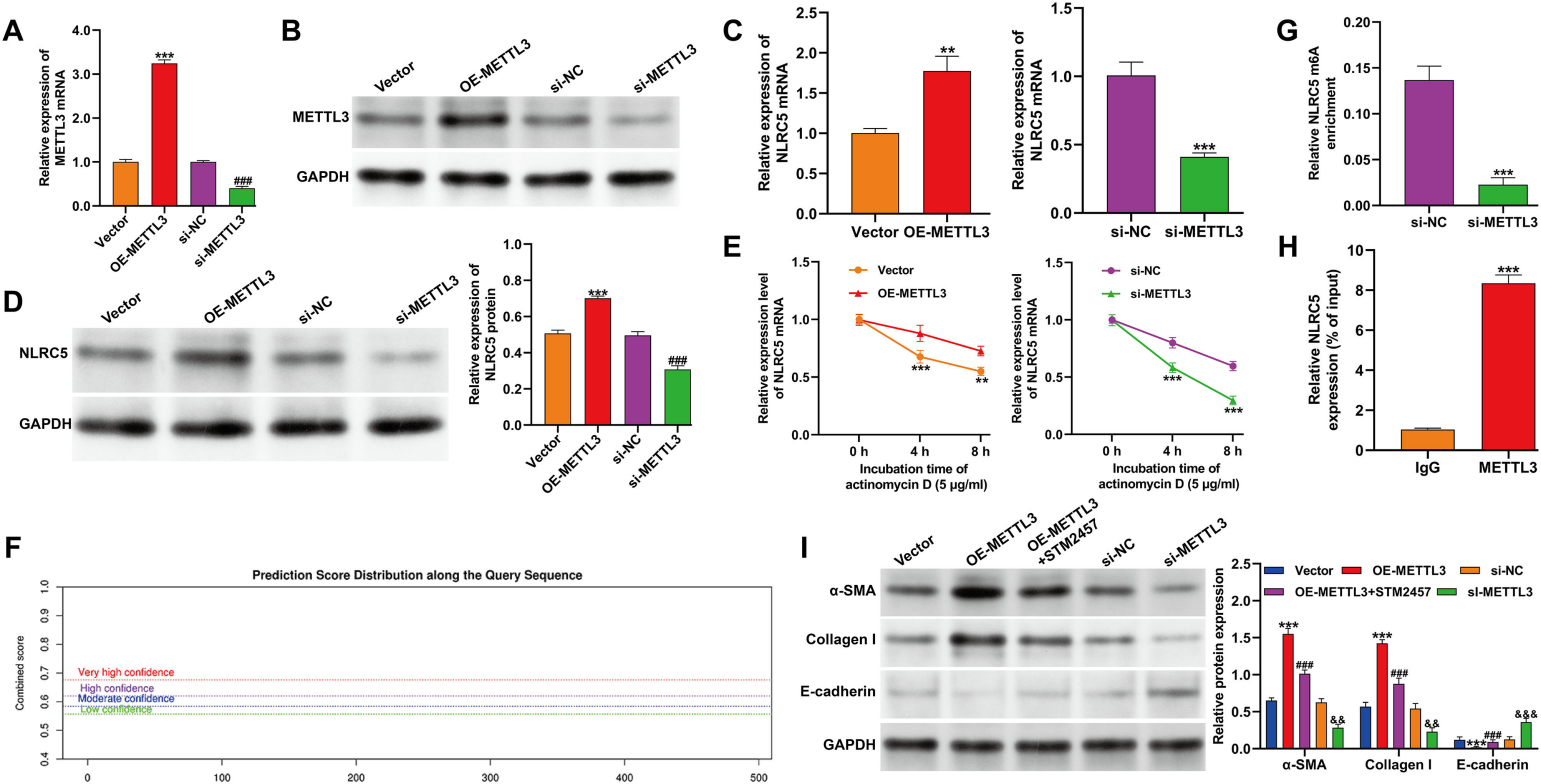
METTL3 regulated m^6^A modification and expression of NLRC5 in TGF-β1-induced cell fibrosis model. **(A)** Quantitative reverse transcription PCR analysis of METTL3 mRNA levels in HK-2 cells transfected with OE-METTL3 or si-METTL3, followed by TGF-β1 (10 ng/mL) treatment for 48 h (***p < 0.001, *vs.* Vector; ###*p* < 0.001, *vs.* si-NC). **(B)** Western blot confirming METTL3 protein expression in the same conditions as **(A, C)** Quantitative reverse transcription PCR showing NLRC5 mRNA levels after METTL3 overexpression or knockdown (**p < 0.01, ***p < 0.001, *vs.* vector or si-NC). **(D)** Western blot analysis of NLRC5 protein expression under the indicated conditions (***p < 0.001, *vs.* vector; ^###^*p* < 0.001, *vs.* si-NC). **(E)** NLRC5 mRNA stability assessed by actinomycin D chase assay in METTL3-modulated cells, measured by quantitative reverse transcription PCR at 0, 4, and 8 h. **(F)** Predicted m6A methylation sites in the NLRC5 transcript analyzed by SRAMP. The graph illustrates the distribution of m6A sites across the transcript, with confidence levels indicated by different color thresholds. **(G)** MeRIP-qPCR showing reduced m^6^A enrichment on NLRC5 mRNA upon METTL3 knockdown. **(H)** RIP-qPCR analysis indicating direct interaction between METTL3 and NLRC5 mRNA, with IgG as control (**p < 0.01, ***p < 0.001, *vs.* indicated controls). **(I)** Western blot of fibrosis markers (α-SMA, Collagen I, and E-cadherin) following METTL3 overexpression or knockdown, with or without STM2457 treatment (***p < 0.001, *vs.* vector; ^###^*p* < 0.001, *vs.* OE-METTL3; ^&&^*p* < 0.01, ^&&&^*p* < 0.001, *vs.* si-NC). Data are presented as mean ± SD from three independent experiments.

The concurrent elevation of METTL3 and NLRC5 expression in TGF-β1–treated HK-2 cells suggested that METTL3 may regulate NLRC5 through m^6^A-dependent mechanisms. To test this hypothesis, METTL3 was either overexpressed (OE-METTL3) or silenced (si-METTL3) in TGF-β1–stimulated cells. Efficient modulation of METTL3 was confirmed at both mRNA and protein levels by qRT-PCR and Western blot analyses (**Figures 2A, B**). NLRC5 expression positively correlated with METTL3 levels, being significantly increased upon METTL3 overexpression and decreased following METTL3 knockdown (**Figures 2C, D**). RNA stability assays further demonstrated that METTL3 enhanced NLRC5 mRNA stability, whereas its silencing accelerated transcript degradation (**Figure 2E**). To investigate potential m6A modification sites, the SRAMP online tool was used to predict high-confidence m6A motifs within the NLRC5 transcript (**Figure 2F**). Consistent with these predictions, MeRIP-qPCR analysis revealed that METTL3 depletion significantly reduced m6A enrichment on NLRC5 mRNA (**Figure 2G**). RIP-qPCR further confirmed a direct physical interaction between METTL3 and NLRC5 transcripts (**Figure 2H**), supporting a mechanism in which METTL3 directly mediates NLRC5 m6A modification. Functionally, METTL3 knockdown attenuated the expression of fibrotic markers α-SMA and Collagen I while restoring E-cadherin levels, whereas METTL3 overexpression produced the opposite effects. Treatment with the METTL3 inhibitor STM2457 effectively reversed the pro-fibrotic changes induced by METTL3 overexpression (**Figure 2I**), indicating that the regulatory effect of METTL3 depends on its methyltransferase activity. Together, SRAMP prediction combined with MeRIP, RIP, RNA stability, and inhibitor assays indicates that METTL3 enhances NLRC5 expression via m6A-dependent mRNA stabilization, contributing to TGF-β1–induced fibrotic responses in HK-2 cells. Despite the absence of site-directed mutagenesis, the integrated evidence supports an m6A-dependent regulatory role of METTL3 on NLRC5.

### Knockdown of NLRC5 exerted suppressive effects in TGF-β1-induced cell fibrosis model

**Figure 3:**
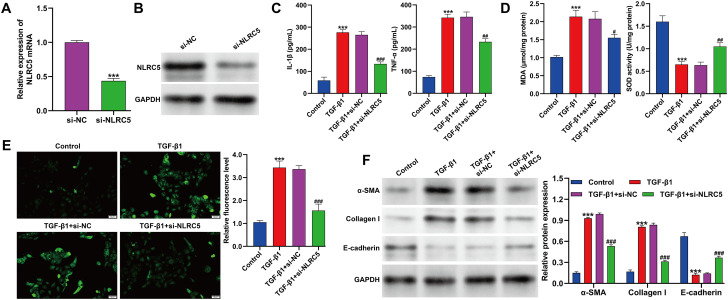
Effects of NLRC5 knockdown on inflammation, oxidative stress, and fibrosis in TGF-β1–treated HK-2 cells. **(A, B)** Quantitative reverse transcription PCR **(A)** and Western blot analysis **(B)** confirming the efficient knockdown of NLRC5 expression in TGF-β1–stimulated HK-2 cells transfected with si-NLRC5 (***p < 0.001, *vs.* si-NC). **(C)** ELISA detection of IL-1β and TNF-α levels in cell culture supernatants following TGF-β1 treatment and/or NLRC5 silencing. **(D)** Measurement of oxidative stress indicators, including MDA content and SOD activity, under different experimental conditions. **(E)** Representative fluorescence images and quantitative analysis of intracellular ROS levels detected using the DCFDA probe in the indicated groups. **(F)** Western blot analysis of fibrosis-related proteins, including α-SMA, Collagen I, and E-cadherin, in TGF-β1–treated HK-2 cells with or without NLRC5 knockdown (***p < 0.001, *vs.* control; ^#^*p* < 0.05, ^##^*p* < 0.01, ^###^*p* < 0.001, *vs.* TGF-β1 + si-NC). Data are presented as mean ± SD from three independent experiments.

To elucidate the function of NLRC5 in TGF-β1–mediated renal fibrosis, NLRC5 expression was effectively suppressed by si-NLRC5 transfection in TGF-β1-stimulated HK-2 cells, as confirmed by quantitative reverse transcription PCR (**Figure 3A**) and Western blot analysis (**Figure 3B**). ELISA results demonstrated that NLRC5 knockdown markedly reduced the secretion of proinflammatory cytokines IL-1β and TNF-α (**Figure 3C**). Moreover, the increased MDA level and decreased SOD activity induced by TGF-β1 were significantly restored upon NLRC5 silencing (**Figure 3D**). Consistently, DCFDA fluorescence analysis revealed that NLRC5 depletion mitigated TGF-β1–triggered ROS accumulation (**Figure 3E**). Finally, Western blot analysis showed that NLRC5 knockdown suppressed the TGF-β1–induced upregulation of α-SMA and Collagen I while reversing the reduction of E-cadherin expression (**Figure 3F**). Collectively, these findings indicate that NLRC5 silencing alleviates TGF-β1–induced inflammatory, oxidative, and fibrotic responses in kidney tubular epithelial cells.

### Keap1/Nrf2/ARE signaling mediated the anti-fibrotic and antioxidative effects of NLRC5 silencing in TGF-β1–induced HK-2 cells

**Figure 4:**
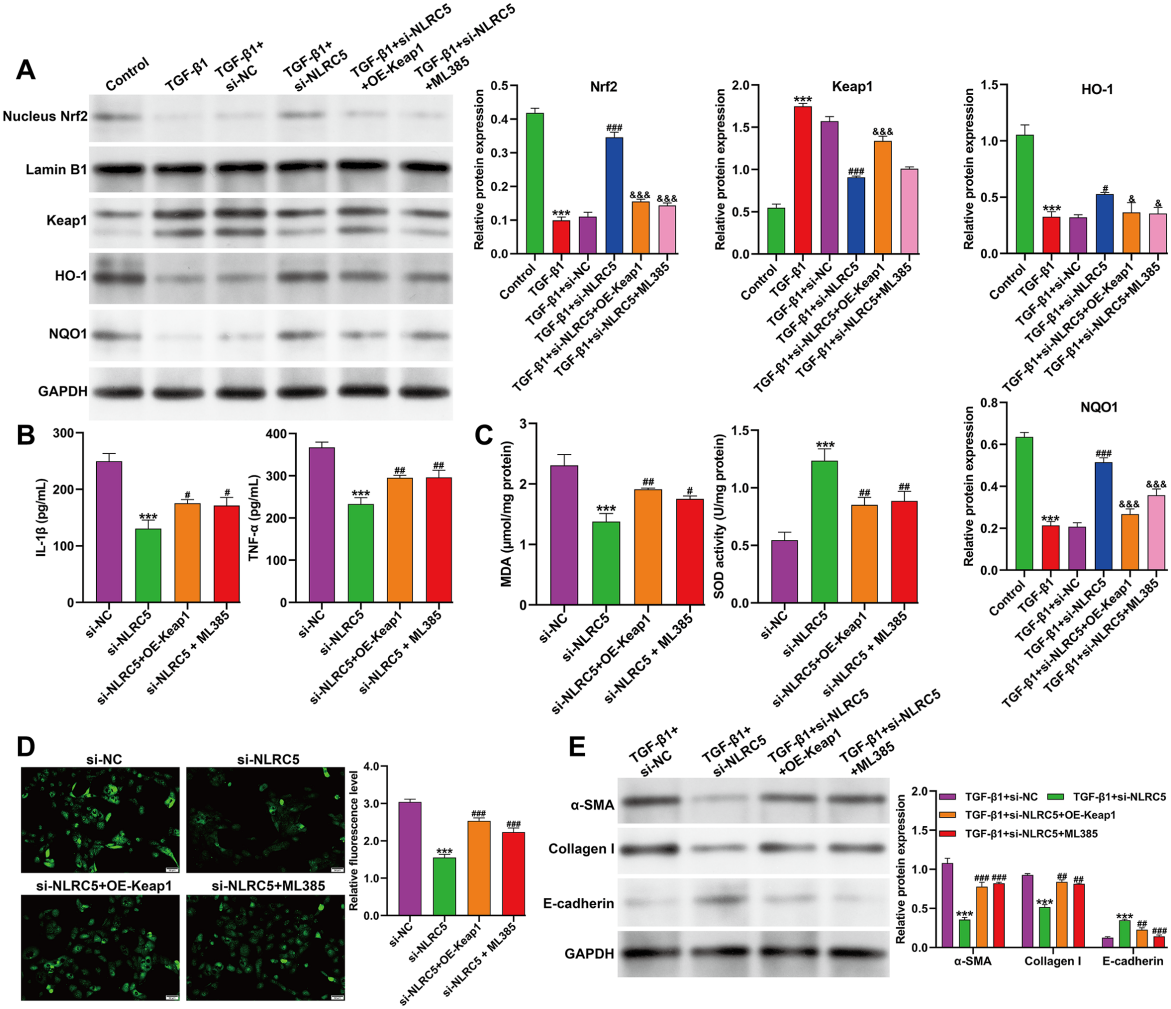
Involvement of the Keap1/Nrf2/ARE signaling pathway in the regulatory effects of NLRC5 knockdown in TGF-β1–induced HK-2 cells. **(A)** Western blot analysis of Keap1, nuclear Nrf2, and the Nrf2 downstream targets HO-1 and NQO-1 in HK-2 cells under the indicated conditions (Control, TGF-β1, TGF-β1 + si-NC, TGF-β1 + si-NLRC5, TGF-β1 + si-NLRC5 + OE-Keap1, and TGF-β1 + si-NLRC5 + ML385). Statistical significance is indicated as follows: ***p < 0.001, *vs.* control; ^#^*p* < 0.05, ^###^*p* < 0.001, *vs*. TGF-β1 + si-NC; ^&^*p* < 0.05, ^&&&^*p* < 0.001, *vs*. TGF-β1 + si-NLRC5. **(B)** ELISA determination of IL-1β and TNF-α levels in cell culture supernatants following the indicated treatments (***p < 0.001, *vs.* si-NC; ^#^*p* < 0.05, ^##^*p* < 0.01, *vs.* si-NLRC5). **(C)** Quantitative analysis of malondialdehyde (MDA) content and superoxide dismutase (SOD) activity in different groups (***p < 0.001, *vs.* si-NC; ^#^*p* < 0.05, ^##^*p* < 0.01, *vs.* si-NLRC5). **(D)** Representative fluorescence images and quantitative measurement of intracellular reactive oxygen species (ROS) using the DCFDA probe (***p < 0.001, *vs.* si-NC; ^###^*p* < 0.001, *vs.* si-NLRC5). **(E)** Western blot analysis of fibrosis-associated proteins, including α-SMA, Collagen I, and E-cadherin, in HK-2 cells under the indicated conditions (***p < 0.001, *vs.* TGF-β1 + si-NC, ^##^*p* < 0.01, ^###^*p* < 0.001, *vs.* TGF-β1 + si-NLRC5). Data are presented as mean ± SD from three independent experiments.

To further elucidate the mechanism underlying the protective effects of NLRC5 knockdown, we examined the involvement of the Keap1/Nrf2/ARE signaling pathway in TGF-β1–induced HK-2 cells. As shown in **Figure 4A**, Western blot analysis revealed that TGF-β1 stimulation markedly reduced the nuclear expression of Nrf2 as well as its downstream antioxidant enzymes HO-1 and NQO-1, while increasing Keap1 levels. Silencing NLRC5 effectively reversed these changes, as indicated by elevated nuclear Nrf2, HO-1, and NQO-1 expression, along with decreased Keap1 protein levels. However, co-treatment with either Keap1 overexpression (OE-Keap1) or the Nrf2 inhibitor ML385 abrogated the effects of NLRC5 silencing, leading to reduced Nrf2 activation. Consistent with these molecular changes, ELISA results demonstrated that the reduction of IL-1β and TNF-α secretion observed in the si-NLRC5 group was reversed by Keap1 overexpression or ML385 treatment (**Figure 4B**). Similarly, oxidative stress analyses showed that MDA levels decreased and SOD activity increased following NLRC5 knockdown, whereas both parameters returned toward pro-oxidative states upon OE-Keap1 or ML385 treatment (**Figure 4C**). DCFDA fluorescence assays further confirmed that ROS accumulation was markedly reduced in si-NLRC5–treated cells but re-elevated when Keap1 was overexpressed or Nrf2 was inhibited (**Figure 4D**). Moreover, Western blot analysis of fibrosis-related proteins revealed that NLRC5 silencing suppressed the expression of α-SMA and Collagen I while enhancing E-cadherin levels, indicating an attenuation of fibrotic activity. These effects were largely abolished in the presence of OE-Keap1 or ML385, which restored α-SMA and Collagen I expression and reduced E-cadherin (**Figure 4E**). Collectively, these findings suggest that NLRC5 knockdown exerts anti-fibrotic, anti-inflammatory, and antioxidative effects through activation of the Keap1/Nrf2/ARE signaling pathway.

### METTL3 knockdown mitigated TGF-β1–induced fibrosis via NLRC5-dependent modulation of Keap1/Nrf2/ARE signaling

**Figure 5:**
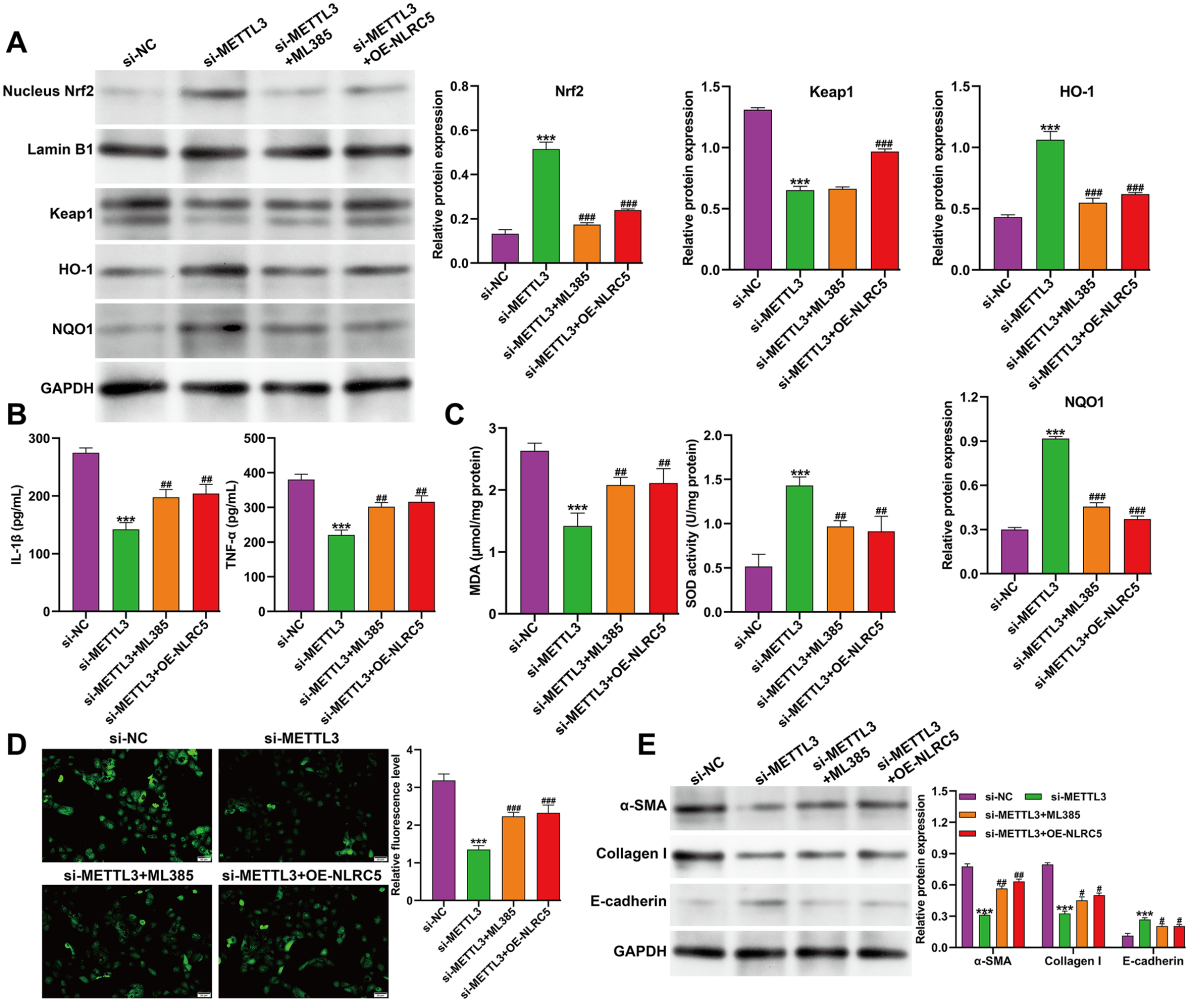
Effects of METTL3 knockdown and NLRC5 modulation on Keap1/Nrf2/ARE signaling and fibrotic responses in TGF-β1–induced HK-2 cells. **(A)** Western blot analysis of Keap1, nuclear Nrf2, and Nrf2 downstream targets HO-1 and NQO-1 in TGF-β1–stimulated HK-2 cells transfected with si-NC, si-METTL3, si-METTL3 + ML385, or si-METTL3 + OE-NLRC5. **(B)** ELISA analysis of IL-1β and TNF-α levels in the culture supernatants of the indicated groups. **(C)** Quantitative assessment of oxidative stress markers, including MDA content and SOD activity, under the indicated treatments. **(D)** Representative fluorescence images and quantitative analysis of intracellular ROS levels detected by the DCFDA probe. **(E)** Western blot analysis of fibrosis-related proteins, including α-SMA, Collagen I, and E-cadherin, in TGF-β1–treated HK-2 cells transfected as indicated. Data are presented as mean ± SD from three independent experiments. ***p < 0.001, versus si-NC; ^#^*p* < 0.05, ^##^*p* < 0.01, ^###^*p* < 0.001, versus si-METTL3.

To further explore whether METTL3 regulates fibrotic progression through the NLRC5–Keap1/Nrf2/ARE axis, TGF-β1–induced HK-2 cells were transfected with si-METTL3 alone or in combination with the Nrf2 inhibitor ML385 or NLRC5 overexpression plasmid (OE-NLRC5). As shown in **Figure 5A**, Western blot analysis revealed that METTL3 knockdown in TGF-β1–stimulated HK-2 cells markedly increased nuclear Nrf2 levels and upregulated its downstream antioxidant targets HO-1 and NQO1, while reducing Keap1 protein. These effects were attenuated by either ML385 treatment or NLRC5 overexpression, which decreased Nrf2, HO-1, and NQO1 levels. To further validate the directional relationship of the NLRC5→Keap1/Nrf2 axis, we performed a complementary experiment in TGF-β1–induced HK-2 cells with NLRC5 overexpression. Western blot confirmed successful NLRC5 overexpression (**Supplementary Figure 1A**). Compared with vector control, OE-NLRC5 cells exhibited decreased nuclear Nrf2 accumulation and reduced HO-1 and NQO1 expression, accompanied by increased Keap1 protein (**Supplementary Figure 1B**), supporting that NLRC5 negatively regulates the Keap1/Nrf2/ARE pathway under fibrotic conditions. Consistent with these findings, ELISA assays showed that the si-METTL3 group exhibited significantly reduced secretion of IL-1β and TNF-α compared with the si-NC group, whereas co-treatment with ML385 or OE-NLRC5 increased cytokine levels (**Figure 5B**). Similarly, oxidative stress markers showed that MDA content was decreased and SOD activity was elevated in METTL3-silenced cells; both changes were reversed by ML385 or NLRC5 overexpression (**Figure 5C**). DCFDA fluorescence analysis confirmed that intracellular ROS generation was suppressed by METTL3 knockdown but re-elevated following either ML385 treatment or NLRC5 overexpression (**Figure 5D**). Furthermore, Western blot analysis of fibrotic markers demonstrated that α-SMA and Collagen I expression were reduced and E-cadherin expression was increased in the si-METTL3 group, indicating attenuation of fibrotic responses. In contrast, ML385 treatment or NLRC5 overexpression reversed these effects, restoring α-SMA and Collagen I levels while suppressing E-cadherin (**Figure 5E**). Together, these results indicate that METTL3 knockdown attenuates TGF-β1–induced inflammation, oxidative stress, and fibrosis in HK-2 cells by activating the Keap1/Nrf2/ARE pathway through suppression of NLRC5.

### METTL3 knockdown alleviated UUO-induced renal injury and inflammation accompanied by NLRC5 downregulation

**Figure 6:**
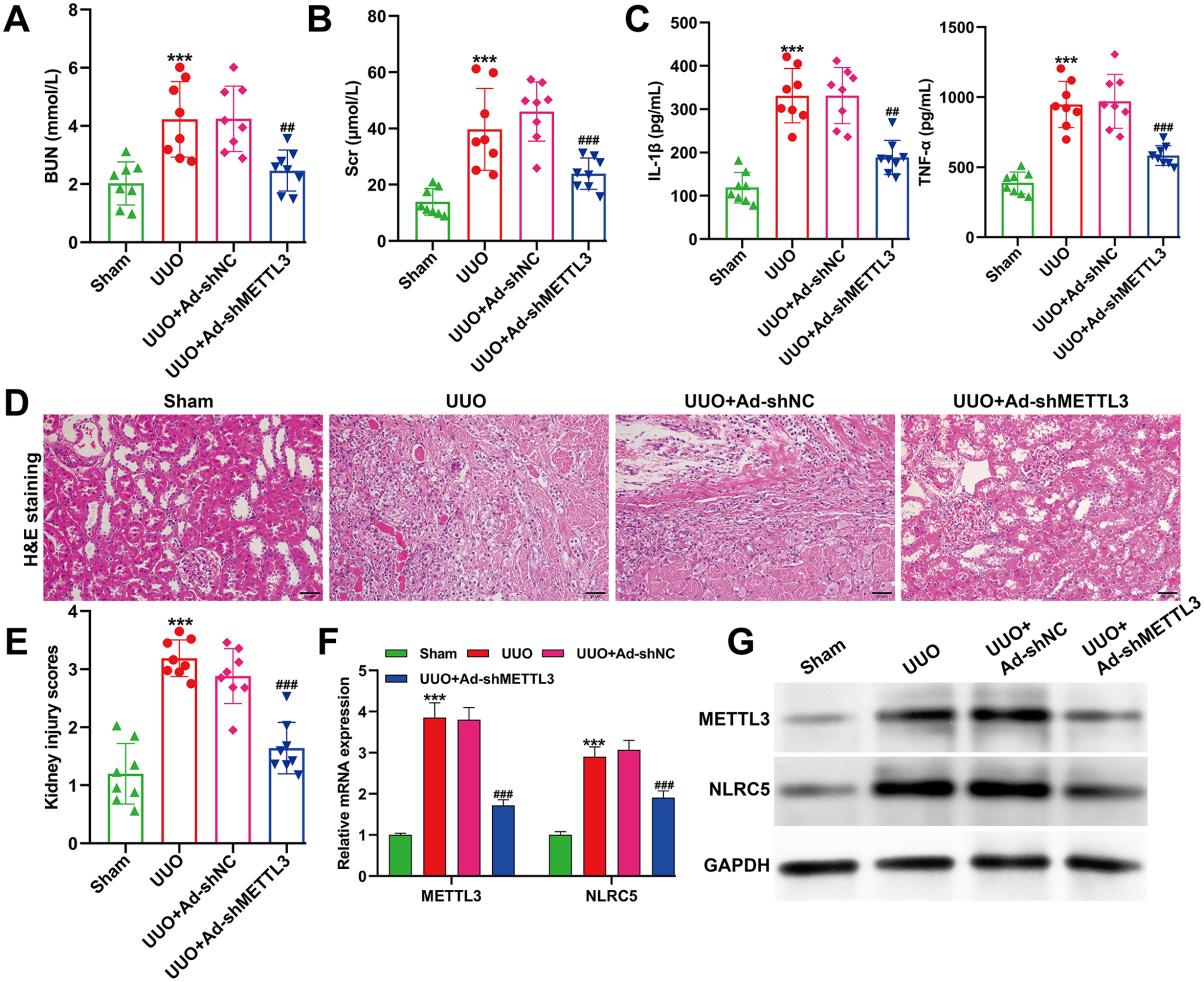
Effects of METTL3 knockdown on renal injury, inflammation, and NLRC5 expression in UUO mice. **(A, B)** Serum levels of blood urea nitrogen (BUN) **(A)** and creatinine (Scr) **(B)** in the sham, UUO, UUO + Ad-sh-NC, and UUO + Ad-shMETTL3 groups. **(C)** ELISA analysis of serum IL-1β and TNF-α levels in mice from the indicated groups. **(D, E)** Representative hematoxylin and eosin (H&E) staining images **(D)** and corresponding semiquantitative histopathological scores **(E)** of kidney tissues, showing renal tubular dilation, epithelial degeneration, and inflammatory cell infiltration in UUO mice, which were markedly alleviated in METTL3-silenced mice. Scale bar = 50 μm. **(F)** Quantitative reverse transcription PCR analysis of METTL3 and NLRC5 mRNA expression in kidney tissues from each group. **(G)** Western blot analysis of METTL3 and NLRC5 protein levels in kidney tissues. Data are presented as mean ± SD (n = 8 mice per group). ***p < 0.001, versus sham; ^##^*p* < 0.01, ^###^*p* < 0.001, versus UUO + Ad-shNC.

To investigate the *in vivo* role of METTL3 in renal fibrosis, a UUO mouse model was established, and animals were divided into four groups: sham, UUO, UUO + Ad-sh-NC, and UUO + Ad-shMETTL3. As shown in **Figures 6A, B**, the levels of BUN and Scr were markedly elevated in UUO mice compared with the sham group, indicating significant renal dysfunction. These elevations were notably reduced following METTL3 silencing. ELISA assays of mouse serum further revealed that proinflammatory cytokines IL-1β and TNF-α were significantly increased in the UUO and UUO + Ad-sh-NC groups but were markedly decreased in the Ad-shMETTL3–treated group (**Figure 6C**). H&E staining showed that the UUO group exhibited prominent tubular dilatation, epithelial cell degeneration and necrosis, and dense inflammatory cell infiltration. In contrast, these pathological alterations were substantially mitigated in METTL3-silenced mice (**Figures 6D, E**). At the molecular level, quantitative reverse transcription PCR (**Figure 6F**) and Western blot analyses (**Figure 6G**) demonstrated that both METTL3 and NLRC5 expression were significantly upregulated in UUO kidney tissues compared with the sham group, whereas METTL3 knockdown led to a concomitant reduction of both genes. Together, these results indicate that suppression of METTL3 alleviates UUO-induced renal injury and inflammation, which is associated with decreased NLRC5 expression.

### Knockdown of METTL3 attenuated renal fibrosis and oxidative stress by activating the Keap1/Nrf2/ARE signaling pathway *in vivo*

**Figure 7:**
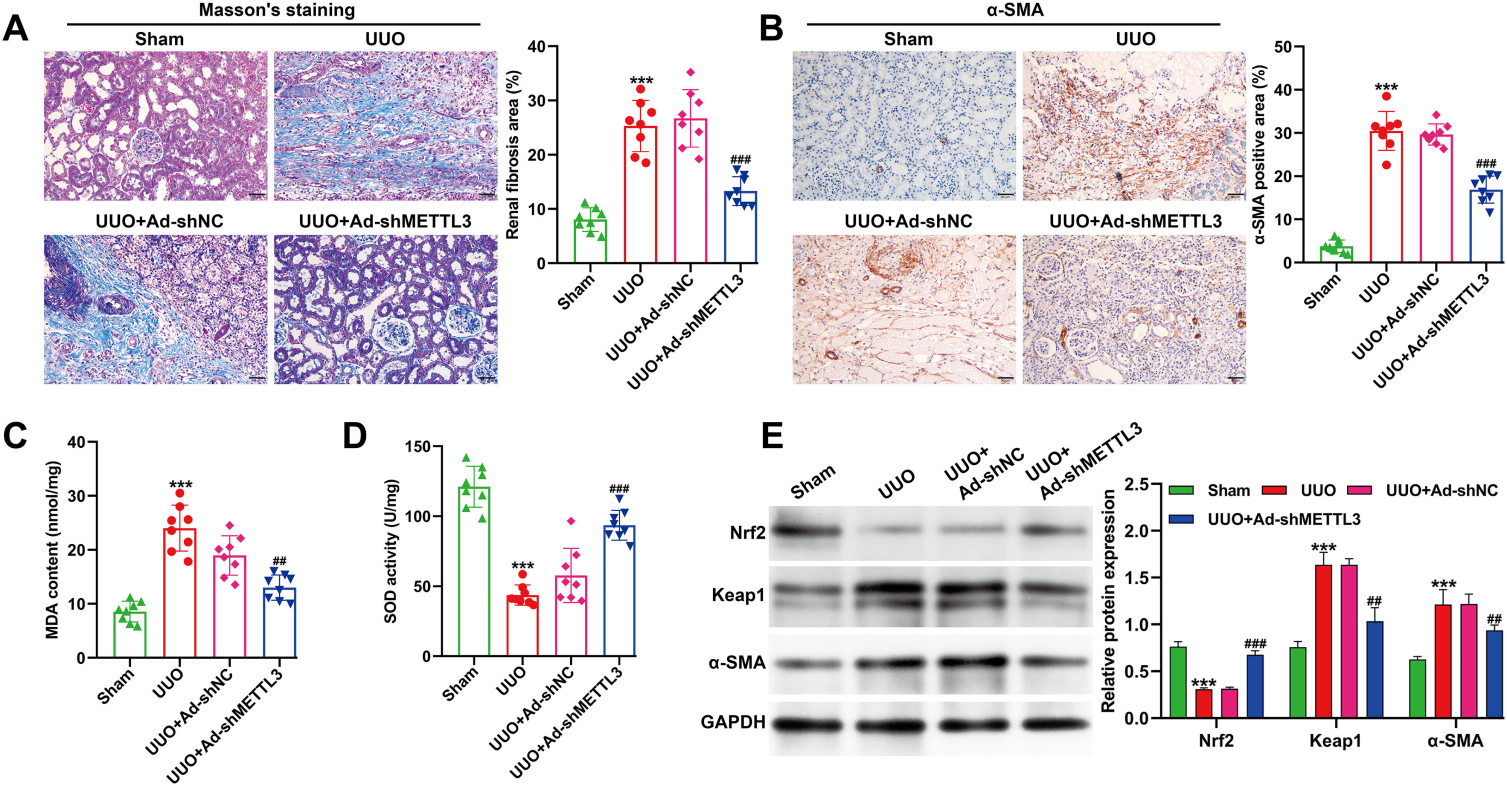
Effects of METTL3 knockdown on UUO-induced renal fibrosis, oxidative stress and the Keap1/Nrf2/ARE pathway in UUO mice. **(A)** Masson’s trichrome staining showing collagen deposition around renal tubules in different groups (n = 8) and quantitative analysis of fibrosis area. Scale bar = 100 μm. **(B)** Immunohistochemical staining of α-SMA in renal cortex tissues. Brown staining represents α-SMA–positive cells. Scale bar = 50 μm. Serum levels of oxidative stress markers, including MDA **(C)** and SOD **(D)**, in each group, assessed by biochemical assays. **(E)** Western blot analysis and quantification of Nrf2, Keap1, α-SMA, and Collagen I protein expression in kidney tissues. Data are presented as mean ± SD. Statistical analysis was performed using one-way ANOVA with Tukey’s test (n = 8 in each group). ***p < 0.001, versus sham; ^##^*p* < 0.01, ^###^*p* < 0.001, versus UUO + Ad-shNC.

Building on the observation that METTL3 knockdown mitigated renal injury and inflammation, we next examined its effects on renal fibrosis and oxidative stress in the UUO model. Mice were assigned to four groups: sham, UUO, UUO + Ad-sh-NC, and UUO + Ad-shMETTL3 (n = 8 per group). Masson’s trichrome staining revealed extensive collagen deposition around renal tubules in the UUO and UUO + Ad-sh-NC groups, indicating marked fibrotic remodeling. In contrast, METTL3 silencing markedly reduced the accumulation of blue-stained collagen fibers, suggesting attenuation of fibrosis (**Figure 7A**). Consistently, immunohistochemical analysis demonstrated that α-SMA, a myofibroblast marker, was strongly induced in the renal cortex following UUO surgery, whereas its expression was markedly suppressed in METTL3-deficient mice (**Figure 7B**). To assess oxidative stress status, MDA and SOD levels were measured in mouse serum. As expected, UUO markedly elevated MDA content while reducing SOD activity, indicating enhanced oxidative injury. Notably, these changes were significantly reversed in the Ad-shMETTL3–treated group (**Figures 7C, D**). Western blot analysis revealed that UUO downregulated Nrf2 protein expression and upregulated Keap1, α-SMA, and Collagen I levels, consistent with oxidative stress and fibrotic activation. Conversely, METTL3 knockdown restored Nrf2 expression and reduced Keap1, α-SMA, and Collagen I levels, indicating reactivation of the Keap1/Nrf2/ARE signaling pathway (**Figure 7E**). Collectively, these findings demonstrate that METTL3 silencing not only alleviates renal inflammation and injury but also suppresses fibrosis and oxidative stress, likely through activation of the Keap1/Nrf2/ARE antioxidant defense pathway.

## Discussion

Growing evidence indicates that aberrant m^6^A modifications play a crucial role in renal pathophysiology, contributing to fibrosis, tubular epithelial injury, and age-related degenerative changes within the kidney (31–34). Consistent with these observations, our data show that TGF-β1 stimulation elevates global m^6^A methylation and upregulates METTL3 expression in HK-2 cells, accompanied by increased expression of fibrotic markers. These findings suggest that m^6^A methylation functions as an epigenetic driver of renal fibrosis, potentially by enhancing the stability of transcripts encoding key pro-fibrotic regulators. Among these, NLRC5 emerges as a critical downstream effector of METTL3. Our *in vitro* and *in vivo* results demonstrate that METTL3-mediated m^6^A modification stabilizes NLRC5 mRNA, thereby linking epi-transcriptomic regulation to inflammatory and fibro-genic signaling.

Notably, it is worth considering whether the METTL3–NLRC5 axis exhibits tissue- or stage-specific regulation. Differential expression of METTL3, NLRC5, or m^6^A reader proteins across tubular, glomerular, or interstitial compartments may contribute to context-dependent outcomes in CKD progression. Previous studies have identified NLRC5 as a multifunctional regulator of fibrosis that integrates immune, inflammatory, and stress-responsive pathways. For example, NLRC5 knockdown has been shown to attenuate renal fibroblast activation and extracellular matrix deposition by suppressing TGF-β1/Smad signaling (17), while its silencing in cardiac tissue alleviates fibrosis through inhibition of the TGF-β1/Smad3 cascade (35). Conversely, NLRC5 overexpression has been implicated in promoting renal inflammation and oxidative stress via activation of the NF-κB and PI3K/Akt signaling pathways, thereby aggravating ischemia–reperfusion and obstructive renal injury (18, 36). These results, together with our current findings, suggest that METTL3-induced stabilization of NLRC5 mRNA may amplify fibro-genic signaling and oxidative stress responses, thereby driving CKD progression.

Beyond transcript stability, m^6^A modification may influence NLRC5 RNA fate, potentially affecting its subcellular localization, interaction with RNA-binding proteins, or alternative splicing. These additional layers of regulation could diversify NLRC5’s functional impact on fibrotic and inflammatory pathways, offering mechanistic explanations for the context-dependent effects observed in CKD. In our study, NLRC5 knockdown alleviated TGF-β1–induced ROS accumulation, restored antioxidant defenses, and reduced proinflammatory cytokine secretion, underscoring its contribution to the pathological microenvironment. Beyond its immunoregulatory functions, NLRC5 has been reported to participate in regulation of NF-κB–driven inflammation in hepatic fibrosis models, supporting its role in promoting oxidative and inflammatory responses (37). These findings extend prior knowledge by linking post-transcriptional m6A regulation to immune-mediated and oxidative mechanisms in fibrotic kidney disease.

From a therapeutic perspective, pharmacological inhibition of METTL3 or modulation of NLRC5 demonstrates anti-fibrotic efficacy. METTL3 inhibition has been shown in other disease models to reduce transcript stability of profibrotic and proinflammatory genes (38, 39), and in our cellular assays, STM2457 attenuated fibrotic marker expression, restoring Nrf2-mediated antioxidant responses. While these results support the concept that m^6^A-modifying enzymes may serve as viable intervention points, METTL3’s broad substrate profile raises potential concerns regarding off-target effects. Future studies should evaluate selectivity, tissue specificity, and long-term safety, ideally integrating combinatorial strategies with antioxidant or anti-inflammatory interventions.

Mechanistically, oxidative stress is a well-established driver of CKD progression, and the Keap1/Nrf2/ARE signaling axis constitutes the principal endogenous defense mechanism against ROS−induced injury and subsequent fibrotic remodeling (40). Nrf2, normally sequestered by Keap1 and targeted for proteasomal degradation, translocates to the nucleus under oxidative stress to activate antioxidant genes such as HO−1, NQO1, and GCLC, thereby mitigating ROS accumulation (41). In our study, NLRC5 was identified as a mediator of both fibrotic and oxidative responses, at least in part by suppressing Nrf2 activity. NLRC5 silencing enhanced nuclear Nrf2 accumulation and upregulated downstream antioxidant targets, whereas METTL3 knockdown produced similar protective effects that were reversed by either NLRC5 overexpression or pharmacological inhibition of Nrf2. These findings indicate that METTL3 promotes NLRC5 mRNA stability via m^6^A modification, which in turn indirectly modulates the Keap1/Nrf2/ARE pathway, linking epitranscriptomic regulation to redox homeostasis and fibrotic remodeling. This pathway highlights a potential therapeutic axis, whereby targeting METTL3 or NLRC5 may restore Nrf2-mediated antioxidant defenses and attenuate fibrotic progression.

In conclusion, our study identifies a METTL3–NLRC5–Keap1/Nrf2/ARE axis that contributes to renal fibrosis by coupling m6A-mediated post-transcriptional regulation with oxidative stress and inflammatory responses. Targeting this axis offers a promising therapeutic avenue for mitigating CKD-associated fibrosis.

Several limitations of this study should be addressed. First, the UUO model mainly represents obstructive kidney injury, which may not fully reflect the diversity of CKD causes and stages. Future studies should investigate tissue- and stage-specific roles of the METTL3–NLRC5 axis. Second, while we identified high-confidence m6A sites in NLRC5 and validated METTL3 regulation, site-specific mutagenesis was not performed, and further studies are needed to explore how m6A affects RNA stability, localization, and splicing. Third, other m6A targets likely contribute to fibrosis and should be explored in future studies. Additionally, long-term kidney function after UUO was not assessed, which is important to consider as chronic kidney injury may evolve over time. Finally, while METTL3 and NLRC5 inhibition show promise for reducing fibrosis, potential off-target effects and the broader role of METTL3 in gene regulation should be considered. Long-term studies, including clinical validation, are necessary to confirm the therapeutic potential of targeting this axis.

## Data Availability

The original contributions presented in the study are included in the article/**Supplementary Material**. Further inquiries can be directed to the corresponding author/s.

## Supplementary material

Supplementary Figure 1 NLRC5 overexpression suppresses Keap1/Nrf2/ARE signaling in TGF-β1–induced HK-2 cells. **(A)** Western blot analysis confirming successful overexpression of NLRC5 in TGF-β1–stimulated HK-2 cells. **(B)** Nuclear Nrf2 levels and downstream antioxidant targets HO-1 and NQO1 were measured by Western blot in vector control and OE-NLRC5 cells. Keap1 protein expression was also assessed.
