# Supplementary figures and images for: METTL3-driven m6A modification of NLRC5 promotes renal fibrosis in chronic kidney disease through Keap1/Nrf2/ARE signaling pathway
Source: Front Immunol. 2026 Mar 11;17:1739011. doi: 10.3389/fimmu.2026.1739011 (PMC13014212; doi:10.3389/fimmu.2026.1739011)

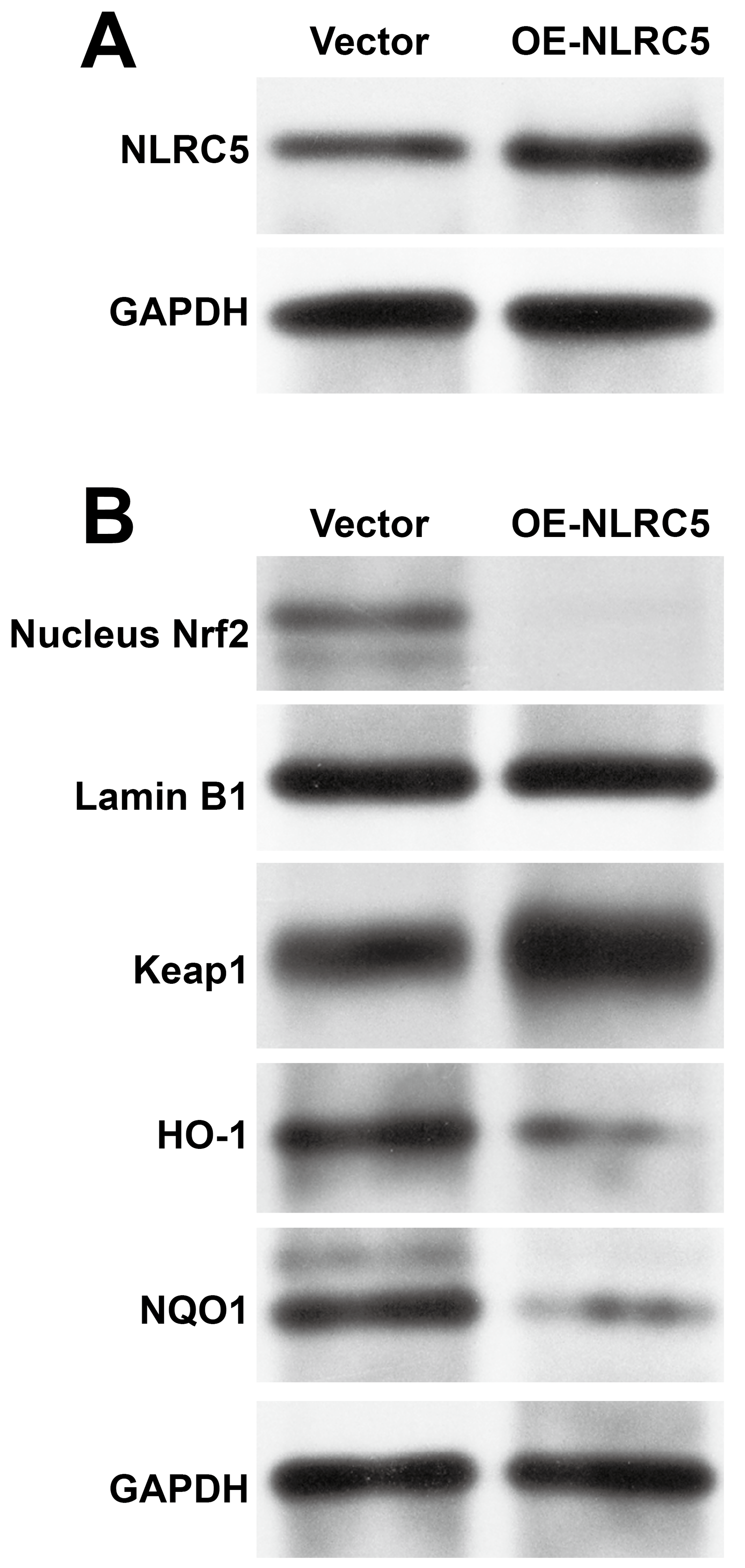

Supplement: Supplementary Figure 1 — NLRC5 overexpression suppresses Keap1/Nrf2/ARE signaling in TGF-β1–induced HK-2 cells. (A) Western blot analysis confirming successful overexpression of NLRC5 in TGF-β1–stimulated HK-2 cells. (B) Nuclear Nrf2 levels and downstream antioxidant targets HO-1 and NQO1 were measured by Western blot in vector control and OE-NLRC5 cells. Keap1 protein expression was also assessed. [file Image1.tif]
